# Expanded HIV Testing in Low-Prevalence, High-Income Countries: A Cost-Effectiveness Analysis for the United Kingdom

**DOI:** 10.1371/journal.pone.0095735

**Published:** 2014-04-24

**Authors:** Elisa F. Long, Roshni Mandalia, Sundhiya Mandalia, Sabina S. Alistar, Eduard J. Beck, Margaret L. Brandeau

**Affiliations:** 1 UCLA Anderson School of Management, Los Angeles, California, United States of America; 2 Co-ordinating and Analytic Centre, National Prospective Monitoring System – HIV Health-economics Collaboration (NPMS-HHC CIC), London, United Kingdom; 3 Department of Management Science and Engineering, Stanford University, Stanford, California, United States of America; 4 Health Services Research and Policy Department, London School of Hygiene and Tropical Medicine, London, United Kingdom; Public Health Agency of Barcelona, Spain

## Abstract

**Objective:**

In many high-income countries with low HIV prevalence, significant numbers of persons living with HIV (PLHIV) remain undiagnosed. Identification of PLHIV via HIV testing offers timely access to lifesaving antiretroviral therapy (ART) and decreases HIV transmission. We estimated the effectiveness and cost-effectiveness of HIV testing in the United Kingdom (UK), where 25% of PLHIV are estimated to be undiagnosed.

**Design:**

We developed a dynamic compartmental model to analyze strategies to expand HIV testing and treatment in the UK, with particular focus on men who have sex with men (MSM), people who inject drugs (PWID), and individuals from HIV-endemic countries.

**Methods:**

We estimated HIV prevalence, incidence, quality-adjusted life years (QALYs), and health care costs over 10 years, and cost-effectiveness.

**Results:**

Annual HIV testing of all adults could avert 5% of new infections, even with no behavior change following HIV diagnosis because of earlier ART initiation, or up to 18% if risky behavior is halved. This strategy costs £67,000–£106,000/QALY gained. Providing annual testing only to MSM, PWID, and people from HIV-endemic countries, and one-time testing for all other adults, prevents 4–15% of infections, requires one-fourth as many tests to diagnose each PLHIV, and costs £17,500/QALY gained. Augmenting this program with increased ART access could add 145,000 QALYs to the population over 10 years, at £26,800/QALY gained.

**Conclusions:**

Annual HIV testing of key populations in the UK is very cost-effective. Additional one-time testing of all other adults could identify the majority of undiagnosed PLHIV. These findings are potentially relevant to other low-prevalence, high-income countries.

## Introduction

Following the recognition that antiretroviral therapy (ART) has both preventive and therapeutic benefits [1], the increased availability of HIV testing programs has enabled persons living with HIV (PLHIV) to receive earlier treatment and care. These programs include “test and treat” [2], “universal access to HIV services” [3] and, for high-prevalence countries, “universal access to HIV and medical male circumcision services” [4]. While most programs focus on high-prevalence countries with generalized epidemics, the underlying principles also apply to countries with concentrated or low-prevalence epidemics, which often contain sizable numbers of PLHIV who are undiagnosed. Studies in the United States and France have demonstrated that more proactive testing policies in low-prevalence, high-income countries, including frequent testing among key populations, are cost-effective compared with current guidelines [5], [6].

In 2011, an estimated 96,000 people (95% credible interval 90,800–102,500) were living with HIV in the United Kingdom (UK), with 24% (19%–28%) unaware of their infection status [7]. Of the 6,280 newly diagnosed HIV infections in 2011 in the UK, 48% were among men who have sex with men (MSM), 2% occurred among people who inject drugs (PWID), and more than half of the remaining heterosexually acquired infections were in black African individuals [7]. HIV incidence among UK MSM has remained stable since 2005 despite high antiretroviral therapy (ART) utilization and only modest increases in reports of condom-less sex [7], [8]. Furthermore, the percentage of heterosexuals who acquired HIV in the UK increased from an estimated 27% in 2002 to 52% in 2011, indicating rising HIV transmission within the UK [7]. These developments highlight the need for further prevention and treatment efforts among MSM and heterosexuals, particularly among black African communities [9] and especially in London [7], [10].

Identifying undiagnosed PLHIV through a voluntary counseling and testing (VCT) program could offer timely access to ART, thereby reducing morbidity and mortality, and could decrease unsafe behaviors [2], [3]. In 2011, 47% of newly diagnosed PLHIV in the UK presented late with a CD4 count <350 cells/mm^3^, and 26% had a CD4 count <200 cells/mm^3^, with disproportionately later diagnoses among heterosexual men and women [7]. The cost-effectiveness of early entry into HIV treatment and care in the UK was recently demonstrated [11]. Additionally, increased ART coverage and earlier ART initiation may reduce transmission to uninfected partners, potentially curbing the country's HIV epidemic [1].

Recent recommendations from the British HIV Association, the British Association for Sexual Health and HIV, and the British Infection Society propose expanding VCT provision to all health settings, and offering routine testing to all adults aged 15–64 years where HIV prevalence exceeds 0.2% [12]. These guidelines have been endorsed by the Health Protection Agency [13] and by the National Institute for Health and Care Excellence (NICE), which recommended a particular focus on testing MSM and people from black African communities [14], [15]. Several pilot HIV testing projects in the UK showed promising initial results, with more than a 60% increase in test uptake in hospital and primary care settings [16].

However, with most HIV testing still concentrated in genitourinary medicine and antenatal clinics [17], concerns have been raised about the UK's ability to tackle its HIV epidemic [18]. The authors of a recent study concluded that *“despite universal access to ART and care in the UK, treatment as prevention is unlikely to decrease HIV transmission at the population level unless the undiagnosed population can be substantially reduced through increasing both the coverage and frequency of HIV testing”* [19].

To date, few costing studies of population-based testing programs in the UK have been performed. A 2011 costing exercise estimated that the cost of detecting a PLHIV in the UK ranged between £2,222 and £3,793 (2010 prices) [20], and the cost of universal HIV testing in the UK was “favourable” when compared with screening programs for other conditions [20]. However, the potential reduction in new HIV infections associated with universal HIV testing in the UK – and its relative cost-effectiveness – has not been examined. In this study, we analyzed the impact of various strategies to expand HIV testing in the UK, based on more recent biomedical and cost data, with a particular focus on MSM and members of black African communities. We evaluated the impact of such expanded policies on the evolution of the HIV epidemic in the UK, and estimated their relative cost-effectiveness.

## Methods

### Overview

We populated a previously published dynamic HIV epidemic model with epidemiological, behavioral, and cost data from the UK. All model parameters are provided in Table 1; model details are published elsewhere [5], [21], [22]. The model simulated HIV transmission in the UK adult population, accounting for varying risk behavior, and projected the future epidemic trajectory under different HIV testing and treatment scale-up scenarios. We then performed a cost-effectiveness analysis to estimate the relative costs and health benefits associated with each scenario.

**Table 1:** Summary of key model parameters.

Variable	Base Value	Range	Source
**Demographic Characteristics**			
***Population of UK residents (adults aged 15–64)***			
People from HIV-endemic countries			
Men	420,000	300,000–500,000	[38]
Women	406,000	300,000–500,000	[38]
PWID (men and women)	200,000	100,000–400,000	[39], [40]
MSM	800,000	600,000–1,200,000	[41]
All others			
Men	30,643,254	-	[42]
Women	31,618,713	-	[42]
***HIV prevalence among UK residents, %***			
People from HIV-endemic countries			
Men	2.5%	2–8%	[43]
Women	5.0%	3–8%	[43]
PWID (men and women)	1.2%	0.6–4%	[40]
MSM	5.0%	3–6%	[44]
All others			
Men	0.033%	0.02–0.05%	[45]
Women	0.033%	0.02–0.05%	[45]
***Annual mortality rate***			
Men	0.00312	0.0030–0.0035	[46]
Women	0.00192	0.0015–0.0025	[46]
PWID (excess)	0.01	0.001–0.02	[40]
***Annual maturation rate out of population***			
Men	0.0188	0.01–0.025	[47]
Women	0.0185	0.01–0.025	[47]
***Annual maturation rate into population***			
Men	0.0261	0.02–0.05	[47]
Women	0.0251	0.02–0.05	[47]
***Annual immigration rate***			
Men	0.025	0.01–0.05	[29]
Women	0.025	0.01–0.05	[29]
***Proportion of new immigrants from HIV endemic countries who are HIV-infected***			
Men	2.5%	2–8%	Assumed
Women	5.0%	3–8%	Assumed
**Sexual Transmission**			
***Transmission probability per partnership***			
Heterosexual (female to male)			
Acute HIV	0.20	0.10–0.30	[48]–[52]
Asymptomatic HIV	0.02	0.01–0.04	[48], [53]–[60]
Symptomatic HIV	0.03	0.01–0.04	[48], [53]–[60]
AIDS	0.05	0.03–0.06	[48], [53]–[60]
Heterosexual (male to female)			
Acute HIV	0.30	0.10–0.40	[48]–[52]
Asymptomatic HIV	0.03	0.02–0.05	[48], [53]–[60]
Symptomatic HIV	0.04	0.02–0.05	[48], [53]–[60]
AIDS	0.08	0.05–0.10	[48], [53]–[60]
Homosexual (male to male)			
Acute HIV	0.40	0.20–0.50	[48]–[52]
Asymptomatic HIV	0.04	0.03–0.06	[61], [62]
Symptomatic HIV	0.05	0.03–0.06	[61], [62]
AIDS	0.10	0.08–0.15	[61], [62]
***Annual number of same-sex partners***			
MSM	4.2	2–10	[63]
***Condom use with same-sex partners, %***			
MSM	56%	25–75%	[8], [64]
***Annual number of opposite-sex partners***			
PWID	3.0	2–5	[65]
All other heterosexuals	1.5	1–2	[66]
***Condom use with opposite-sex partners, %***			
PWID	17%	10–25%	[65]
All other heterosexuals	20%	7–41%	[66]
Fraction of men who are circumcised	16%	5–25%	[67]
Reduction in heterosexual HIV transmission due to male circumcision, %	50%	48–60%	[68]–[70]
**Injection Drug Use Transmission**			
***Transmission probability per shared injection***			
Acute HIV	0.016	0.008–0.040	[48], [49], [51], [71], [72]
Asymptomatic HIV	0.002	0.001–0.005	[55], [72]–[74]
Symptomatic HIV	0.003	0.001–0.005	[55], [72]–[74]
AIDS	0.003	0.001–0.005	[55], [72]–[74]
Average number of injections per year	432	300–564	[75]
Fraction of injections that are shared, %	17%	16–22%	[65]
**Disease Progression (annual rates)**			
***Acute HIV to asymptomatic HIV***	6	4–12	[48]–[50]
***Asymptomatic HIV to symptomatic HIV***			
Untreated	0.13	0.10–0.20	[5], [6]
Treated	0.08	0.05–0.10	[5], [6]
***Symptomatic HIV to AIDS***			
Untreated	0.33	0.20–0.50	[5], [6]
Treated	0.06	0.05–0.10	[5], [6]
***AIDS to death***			
Untreated	0.40	0.25–0.50	[5], [6]
Treated	0.25	0.10–0.50	[5], [6]
**Disease Stages (initial distribution), %**			
Acute HIV	1%	0–5%	Calculated
Asymptomatic HIV	40%	30–50%	[76]
Symptomatic HIV	16%	10–20%	[76]
AIDS	43%	30–50%	[76]
**HIV Testing and Counseling**			
***Fraction of population tested in past 12 months, %***			
High-risk persons			
PWID	77%	50–80%	[65]
MSM	25%	10–50%	[8]
People from HIV-endemic countries	25%	10–60%	[77]
All other persons	10%	5–50%	[16]
***Annual probability of symptom-based case finding, %***			
HIV	10%	0–20%	[5], [6]
AIDS	20%	8–50%	[5], [6]
***Sexual behavior in HIV patients***			
Reduction in sexual behavior among persons identified as HIV-positive, %	25%	0–50%	Assumed
**Antiretroviral therapy (ART)**			
***Fraction starting ART at CD4 <350 cells/mm^3^, %***			
High-risk persons			
PWID	6%	0–20%	[76]
MSM	46%	25–60%	[76]
People from HIV-endemic countries	22%	10–40%	[76]
All other persons			
Men	75%	50–90%	[76]
Women	23%	20–60%	[76]
Annual ART entry rate if CD4 <350 cells/mm^3^, %	37%	0–50%	[76]
Retention in care 12 months after diagnosis, %	86%	50–95%	[7]
Reduction in injection infectivity due to ART, %	50%	25–75%	[23], [71]
Reduction in sexual infectivity due to ART, %	96%	50–99%	[1]
**Quality-of-Life Multipliers**			
Uninfected	1.00	–	[78]
Acute HIV	0.89	0.60–0.95	[79]–[82]
Unidentified asymptomatic HIV	0.91	0.85–0.95	[79]–[82]
Identified asymptomatic HIV at 1 year	0.84	0.80–0.90	[23], [79]–[82]
Identified asymptomatic HIV at 2+ years	0.89	0.85–0.95	[23], [79]–[82]
Unidentified symptomatic HIV	0.79	0.70–0.80	[79]–[82]
Identified symptomatic HIV	0.72	0.70–0.80	[79]–[82]
Symptomatic HIV treated with ART	0.83	0.82–0.87	[79]–[82]
Unidentified AIDS	0.72	0.60–0.75	[79]–[82]
Identified AIDS	0.72	0.60–0.75	[79]–[82]
AIDS treated with ART	0.82	0.82–0.87	[79]–[82]
PWID (multiplier)*	0.90	0.80–1.00	[71], [72], [74]
**Costs (2012 GBP)**			
***Asymptomatic HIV***			
Untreated	£1,862	£1,220–£2,505	[76]
Treated	£7,793	£5,119–£10,467	[76]
***Symptomatic HIV***			
Untreated	£5,447	£3,889–£7,005	[76]
Treated	£9,305	£7,093–£11,517	[76]
***AIDS***			
Untreated	£13,457	£10,163–£16,750	[76]
Treated	£16,892	£13,962–£19,823	[76]
Annual non–HIV-related health care costs	£2,571	£1,950–£3,213	[11]
Annual cost of ART	£14,294	£11,770–£16,140	[83]
Cost of HIV ELISA antibody test	£8	£3–£12	[16]
Cost of behavior counseling/hour**	£36	£26–£46	[84]
Annual cost of ancillary PWID services	£7,700	£3,000–£10,000	[85]
Annual discount rate	3%	0%–5%	[25]

ART = antiretroviral therapy; MSM = men who have sex with men; PWID = people who inject drugs.

* Quality of life for all PWID is multiplied by this factor, across all health states.

** Under current UK guidelines [24], individuals who are screened for HIV are given a pre-test counseling session and a post-test counseling session.

The length of the post-test counseling session depends on the outcome of the HIV test.

### Population Characteristics

The model (schematically illustrated in Figure S1) captured HIV transmission and progression in the adult population aged 15 to 64 in the UK. We divided the population into six groups, distinguished by risk behaviors or country of origin: MSM; PWID; men from HIV-endemic countries with high HIV prevalence; women from HIV-endemic countries; other men; and other women. We further divided these population groups by HIV infection status: uninfected, acute HIV infection, asymptomatic HIV with CD4 count >350 cells/mm^3^, symptomatic HIV with CD4 count 200–350 cells/mm^3^, or AIDS with CD4 count <200 cells/mm^3^; HIV diagnosis status; ART status if infected; and male circumcision status.

Individuals entered the population through maturation or immigration to the UK from HIV-endemic countries. We assumed that 2.5% of male immigrants and 5.0% of female immigrants entering from HIV-endemic countries were HIV-infected, similar to the proportion living in the UK who were HIV-infected. Individuals exited the population due to age maturation or death.

### HIV Transmission and Disease Progression

HIV transmission occurred via sexual contact among MSM, sexual contact between men and women, and needle sharing among PWID. For each type of sexual contact we estimated the annual number of partnerships, rate of condom use, and transmission probability per partnership. For needle sharing contacts, we estimated the annual number of injections, average needle sharing rates, and probability of transmission per shared needle. Each sexual transmission probability depended on the infected person's gender, disease status, and treatment status and the uninfected person's gender and circumcision status, if male. Upon acquiring HIV infection, individuals passed through the disease states at a rate inversely proportional to the time spent in each state [23]. Death could occur from non-HIV causes from any health state, or from HIV infection among individuals who have symptomatic HIV or AIDS.

### HIV Testing and Treatment

Under the status quo scenario, we assumed that 25% of MSM, 25% of people from HIV-endemic countries, 77% of PWID, and 10% of other adults in the population received an HIV test in the last 12 months [8], [16]. HIV-infected persons can also be diagnosed after developing symptoms with CD4 <350 cells/mm^3^ at an annual probability of 10–20%. Once diagnosed with HIV, we assumed that individuals reduced sexual partnerships by 25%, but we varied this from no change up to a 50% reduction. We modeled two HIV testing approaches: a *universal* strategy where all adults were periodically tested every one, two, or three years; and a *targeted* strategy, where MSM, PWID, and people from HIV-endemic countries were tested every year, and all other adults were tested less frequently, either one-time or every two years. We assumed that the testing and counseling procedures would follow current UK guidelines [24].

Current ART initiation rates vary widely, ranging from only 6% of PWID to 46% of MSM and 75% of diagnosed men living with HIV beginning ART at CD4 count <350 cells/mm^3^. Each year thereafter, an additional 37% of diagnosed individuals commence ART at lower CD4 counts. In our model, we considered a scenario where 75% of PLHIV start ART at CD4 count <350 cells/mm^3^, with additional initiation thereafter. Attrition and loss to follow-up also reduce the proportion of HIV-infected people with viral suppression, with 86% adherent after 12 months. Finally, we assumed that effective ART reduced HIV transmission probabilities via needle-sharing (by 50%) and sexual contact (by 96%) [1].

### Health and Economic Outcomes

For each strategy, we projected HIV prevalence and incidence over a 10-year time horizon, and lifetime quality-adjusted life years (QALYs) accrued in the population. We calculated lifetime healthcare costs for all individuals in the population, which depended on gender, HIV infection and treatment status, and injection drug use status, and we included the cost of VCT and ART per person. We estimated HIV infections averted and the incremental cost-effectiveness ratio (ICER) for each strategy. Taking a societal perspective, we considered all health care costs and savings regardless of source or beneficiary [25]. Costs were in 2012 Great Britain pounds (£), and we discounted all costs and QALYs at 3% annually.

### Model Validation

We compared our model-projected outcomes to available data on HIV prevalence, incidence, and diagnosis trends, and found close approximations (Table 2). For example, our model calculated that 3,471 people acquired HIV in 2011, compared to 3,640 estimated by the UK Health Protection Agency [7]. The number of new HIV diagnoses each year, number of PLHIV by risk category, and proportion of diagnosed PLHIV and those receiving ART were also close to reported estimates. For other outcomes, the paucity of data on HIV epidemic trends in the UK precluded us from conducting a more robust model calibration exercise.

**Table 2:** Model validation results under status quo scenario.

Epidemic Outcome (2011)	Model Estimate	Data*
Total annual new HIV infections	3,471	3,640
Total annual new HIV diagnoses	6,125	6,280
Men	4,386	4,470
Women	1,739	1,810
People living with HIV/AIDS		
Men who have sex with men (MSM)	40,000	40,100
People who inject drugs (PWID)	2,400	2,300
Men from HIV-endemic countries	10,500	10,500
Women from HIV-endemic countries	20,300	20,300
All other adults	20,546	20,200
Total	93,746	93,400
HIV-infected people aware of status	71,013	73,400
HIV-infected people receiving ART	59,000	61,510

* Source: Health Protection Agency. HIV in the United Kingdom: 2012 Report [45].

## Results

### Status Quo

**Figure 1:**
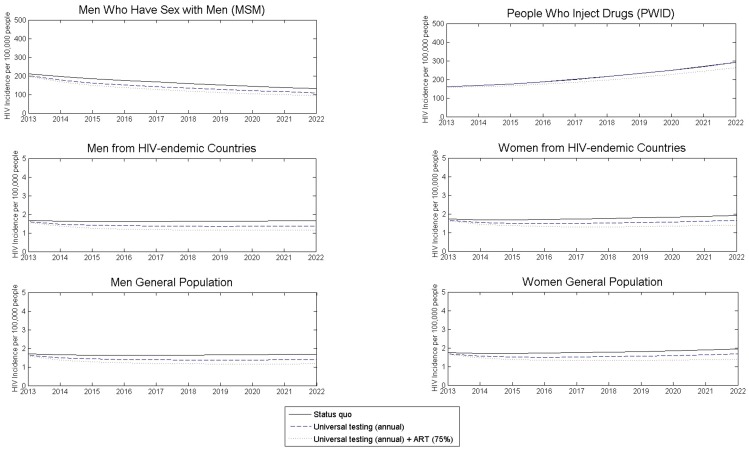
Projected annual HIV incidence over time in the UK under different testing strategies. The six graphs correspond to six different risk groups in the UK, with projected annual HIV incidence per 100,000 people shown under current testing and treatment levels (black solid line), universal annual testing of all adults (blue dashed), or universal annual testing coupled with antiretroviral therapy (ART) initiation of 75% at CD4 <350 cells/mm^3^ (red dotted). The cumulative number of new HIV infections over 10 years is given in Table 3.

If current HIV testing and treatment levels persist, we projected that overall HIV incidence will remain stable at approximately 3,500 new infections per year, and increase slightly by around 3% by year 2022 (Figure 1). More than 6,100 people would be diagnosed with HIV in 2013, including 2,700 MSM, 600 PWID, 2,100 men and women from HIV-endemic countries, and 700 other men and women in the population.

### Universal HIV Testing

**Figure 2:**
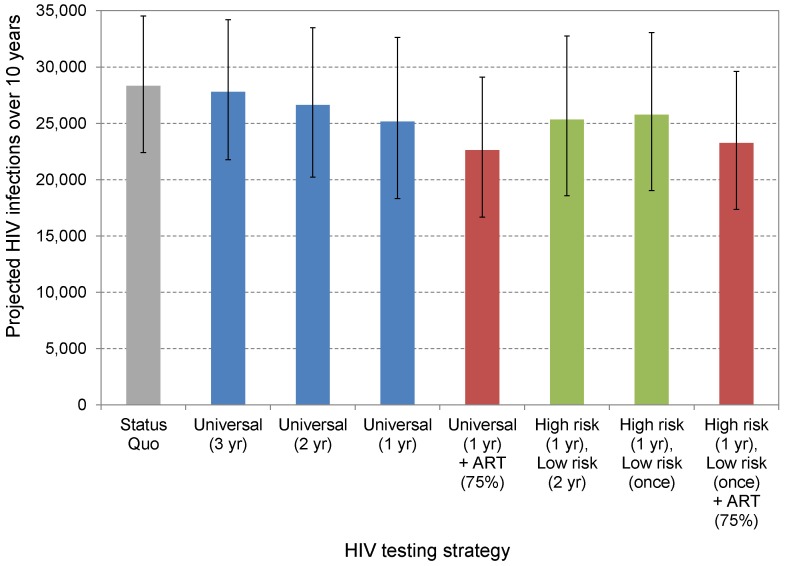
Projected total new HIV infections in the UK (2013–2022) under different HIV testing and treatment strategies. Each bar corresponds to modeled estimates of new HIV infections over the next 10 years, assuming a 25% reduction in sexual partnerships following HIV diagnosis. For each strategy, the higher estimate (top of black line) corresponds to the scenario with no partnership reduction, and the lower estimate (bottom of black line) corresponds to a 50% partnership reduction following diagnosis. The time in parentheses corresponds to the testing interval, and “once” refers to one-time testing of individuals not in the key populations we considered. “ART (75%)” refers to 75% antiretroviral therapy initiation of at CD4 <350 cells/mm^3^.

A comprehensive VCT campaign offered to all adults identified a substantial number of previously undiagnosed PLHIV and prevented a substantial number of new infections, but the aggregate impact depended significantly on the effectiveness of testing and counseling at reducing sexual partnerships (Table 3, Figure 2). Under the most conservative assumption of no partnership reduction, universal VCT averted 1% of new HIV infections with screening every three years or 5% with annual screening, as a consequence of diagnosed PLHIV initiating ART earlier. With a 25% reduction in sexual partnerships following diagnosis, universal testing prevented 2–11% of new infections; a 50% reduction in partnerships decreased new infections by 3–18%. An annual HIV testing program diagnosed 16,000 people in its first year, substantially reducing the number of undiagnosed PLHIV.

**Table 3:** Projected outcomes under different testing strategies.

		HIV Infections (Prevented) over 10 years*					
Scenario	HIV+People Identified over 10 Years	No Reduction in Sexual Behavior	25% Reduction in Sexual Behavior	50% Reduction in Sexual Behavior	Incremental Costs (billions)**	Incremental QALYs**	Incremental Cost/QALY Gained**
Status quo	48,932	34,524	28,324	22,410	–	–	–
Universal testing every 3 years	54,046	(325)	(521)	(639)	£1.25	13,000	£96,200†
Universal testing every 2 years	59,424	(1,032)	(1,705)	(2,181)	£2.18	32,900	£66,300†
Universal testing every 1 year	65,598	(1,887)	(3,180)	(4,081)	£4.61	57,400	£80,300†
Universal testing every 1 year+ART	62,640	(5,411)	(5,709)	(5,749)	£7.41	161,700	£240,000
High-risk testing every 1 year, Low-risk testing every 2 years	64,017	(1,768)	(2,986)	(3,847)	£2.37	53,100	£44,700†
High-risk testing every 1 year, Low-risk testing one time	58,391	(1,461)	(2,555)	(3,368)	£0.75	42,900	£17,500
High-risk testing every 1 year, Low-risk testing one time+ART	56,293	(4,920)	(5,066)	(5,044)	£3.49	145,300	£26,800

* Model-projected new HIV infections in the UK between 2013 and 2022 under status quo testing and treatment levels, and projected number of averted infections with increased testing and treatment in parentheses. Three scenarios for the reduction in risky sexual behavior following HIV diagnosis are given.

** Incremental costs and quality-adjusted life years (QALYs) discounted to the present, are relative to the status quo, assuming a 25% reduction in risky sexual behavior following HIV diagnosis.

† Strategies that are dominated, i.e., have higher costs but generate fewer health benefits than a combination of other strategies.

**Figure 3:**
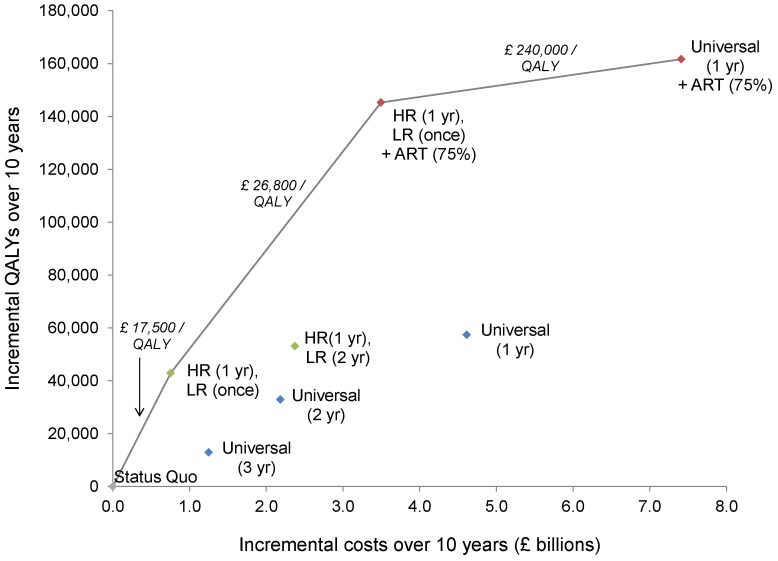
Cost-effectiveness of alternative HIV testing and treatment strategies in the UK. The incremental costs (x-axis) and QALYs (y-axis) of different HIV testing and treatment scenarios are shown, relative to status quo levels. The blue points correspond to universal HIV testing strategies for all adults, with testing every one, two, or three years. The green points correspond to targeted strategies, with annual testing for high-risk persons and testing every two years or one-time for all other persons. The red points correspond to an expanded HIV testing program coupled with 75% antiretroviral therapy initiation of at CD4 <350 cells/mm^3^. The solid black line marks the cost-effectiveness frontier, or the set of strategies that is most economically efficient, and the corresponding incremental cost-effectiveness ratios are given. Costs and QALYs are discounted at 3% annually, and include the direct costs of the programs over 10 years, as well as the lifetime costs and QALYs of all individuals in the population. HR = high-risk, and includes men who have sex with men (MSM), people who inject drugs (PWID), and men and women from HIV-endemic countries. LR = low-risk, and includes men and women who do not belong to the identified key populations. ART = antiretroviral therapy. QALY = quality-adjusted life year.

Although universal HIV testing has the potential to diagnose the greatest number of people and link them to lifesaving treatment, its relative cost-effectiveness may impede its implementation. The population-wide gain in health benefits with annual testing ranged from 46,000–65,000 additional discounted QALYs over 10 years. Our model projected that annual testing of all adults cost £67,400/QALY gained under the optimistic assumption of a 50% reduction in sexual partnerships following diagnosis, and £106,000/QALY gained with no partnership reduction (Figure 3). Each diagnosed PLHIV required on average more than 11,000 people to be tested.

### Targeted HIV Testing

A program that provided annual HIV testing to MSM, PWID, and people from HIV-endemic countries, coupled with one-time screening of all other adults, averted nearly as many infections as universal screening, for a fraction of the cost (Table 3; Figure 3). With this strategy, 4–15% of infections were prevented, depending on behavior change, and more than 15,000 people were diagnosed in the first year. At £17,500/QALY gained, targeted testing was the most cost-effective strategy considered, and required testing fewer than 2,500 people per PLHIV diagnosed.

A slightly more comprehensive program offering repeat HIV testing every two years to lower-risk persons prevented 5–17% of new HIV infections over 10 years. However, the small marginal benefit of repeat testing suggests that a one-time testing program identified most undiagnosed PLHIV not in the key populations we considered; more frequent testing of these individuals only identified people who later acquired HIV. This strategy cost £36,000–£62,000/QALY gained and was more cost-effective than universal testing.

### Expanded ART

The above analyses assumed that ART initiation rates remained at current levels. If ART access increased concomitantly with expanded testing, the health benefits increased dramatically. Universal annual HIV testing with 75% ART initiation for infected individuals with CD4 <350 cells/mm^3^ prevented up to 26% of new HIV infections, adding up to 165,000 QALYs over 10 years at a cost of approximately £240,000/QALY gained, relative to annual testing alone.

If expanded ART was instead linked with targeted HIV testing of high-risk persons every year and one-time testing of all other adults, up to 23% of infections were prevented, adding 148,000 QALYs – or nearly 90% of the benefits accrued with universal testing and ART – at a cost of only £26,800/QALY gained (Figure 3). Under an ideal scenario where ART uptake is 100%, the cost-effectiveness of this strategy remained less than £30,000/QALY gained.

### False Positive and Negative Diagnoses

As with any extensive HIV screening program, the risk of false diagnoses should be considered. Given an HIV testing sequence with 99.5% sensitivity and 99.9994% specificity [5], [21] we estimated that approximately 62 false positives and 350 false negatives would occur in the first year of implementation of annual high-risk and one-time low-risk testing. After 10 years, this dropped to 16 false positives and 65 false negatives per year. False positives decreased because fewer people remained unscreened, and false negatives decreased because fewer PLHIV remained undiagnosed. Of note, MSM, PWID, or men and women from HIV-endemic countries who were falsely diagnosed as uninfected would likely be identified in a subsequent annual screening.

### Sensitivity Analysis

We varied all model parameters in sensitivity analyses (Table 1). Here we highlight key sensitive parameters. A primary driver of health outcomes and cost-effectiveness was the extent to which VCT reduces risky sexual partnerships among newly diagnosed PLHIV. Prior studies have shown reductions as high as 76% in the UK [26], but these were often restricted to the window of acute infection, or included only MSM who were more likely to have many partners before diagnosis. We considered up to a 50% reduction in sexual partnerships across all risk groups: even with this reduction, annual HIV screening of all adults cost nearly £70,000/QALY gained.

The epidemic's baseline trajectory also affected cost-effectiveness estimates, although the relative cost-effectiveness ranking remained unchanged. If HIV prevalence in each risk group was 50% greater than initially assumed, then annual testing of high-risk persons along with one-time testing of all other adults in the population cost £15,000/QALY gained, compared to £17,500 in the base case. With 50% lower prevalence, this strategy exceeded £25,000/QALY gained, because more people must be screened to identify each PLHIV. Similarly, if HIV transmission probabilities were 50% higher than initially assumed, the cost-effectiveness of this strategy improved to £7,000/QALY gained; with 50% lower transmission probabilities, the cost-effectiveness worsened to £36,000/QALY gained. Increased testing and counseling costs, increased annual ART costs, reduced adherence levels, and reduced ART effectiveness all slightly mitigated the benefits of expanded HIV testing, but again the relative cost-effectiveness of the strategies under consideration remained unchanged.

## Discussion

The HIV epidemic in the UK currently remains relatively concentrated among MSM and people born in HIV-endemic countries and related communities, and efforts to identify undiagnosed PLHIV and link them to care have been insufficient. With one-quarter of PLHIV unaware of their HIV status, and approximately 3,500 people continuing to acquire HIV every year, the need for improved HIV testing and counseling is evident. Who to test – and how frequently – had not yet been systematically evaluated in the UK. Our study addresses these important policy questions and provides strong support for increased HIV testing of adults.

Our analysis suggests that periodic testing of all adults in the UK, regardless of risk status, could prevent 18% of new infections with existing treatment levels, or nearly 30% of new infections if treatment levels increase simultaneously. Nevertheless, universal testing is less efficient than targeted testing, requiring four times as many tests to identify each PLHIV. A targeted testing approach that offers annual HIV testing to MSM, PWID, and people from HIV-endemic countries, along with one-time screening of all other adults, could offer 80% of the benefits of universal testing for only 14% of the cost over 10 years. At £17,500/QALY gained, this strategy is well below established UK cost-effectiveness thresholds of £20,000–£30,000/QALY gained [27], [28]. An important advantage of this strategy is the frequency of testing offered to people from HIV-endemic countries, including the approximately 15,000–20,000 people who emigrate from African countries to the UK each year [29]. Furthermore, expanding access to ART for these key populations helps ensure that they receive timely and effective care.

As with any risk-based disease screening program, a key challenge of the proposed targeted HIV testing strategy is identifying high-risk individuals and incentivizing VCT uptake. An advantage of one-time universal testing of all adults is that it may reduce stigma and reticence among key populations to get tested. Of course, any wide scale VCT program should focus efforts on linking PLHIV to care and ensuring effective counseling aimed at reducing risky sexual behavior following diagnosis.

Our analysis has several limitations. As with many epidemic models, we simplified the complex dynamics of HIV disease progression, development of resistance, and changes in viral suppression. Although our model captured the reduction in primary transmission to the partners of persons diagnosed with HIV, as well as secondary transmission to those partners' partners, we assumed a standard proportional mixing model of partnership selection. Due to data limitations we did not include preferential mixing by HIV status, race or immigration status, nor did we consider differential condom use by HIV status. We assumed similar HIV prevalence levels for newly arriving immigrants and those already living in the UK, due to a lack of data on HIV infection rates of those just arriving. Improved data on baseline demographics, sexual behavior and other risk behaviors would allow for more refined estimates of testing impact. Finally, we estimated costs on a per person basis using current estimates of the costs of HIV testing and counseling and treatment for HIV infection. If expansion of HIV testing coverage were linked with a broad national campaign or with significant changes in delivery of health care services then costs could be higher than we have estimated.

Despite its limitations, in particular the lack of current accurate UK epidemic data, our study showed that targeted HIV testing of specific key populations is cost-effective in the UK. This finding is potentially relevant to other low-prevalence, high-income countries [30]. Implementation should include relevant health professionals and civil society [30]. Implementation of HIV testing programs should occur while maintaining or developing other high-quality HIV prevention and therapeutic services, and needs to include relevant health professionals and community representatives to ensure best practices for the development and implementation of such targeted testing campaigns.

The success of any country's response to its HIV epidemic rests on ‘know your epidemic’ – the key populations that drive the epidemic – and ‘know your response’ – especially interventions that directly involve those populations [31]. A country's HIV prevention program must involve the use of a combination of prevention modalities including biomedical, behavioral and structural interventions [32], deployed in a way that minimizes stigma and discrimination at both personal and community levels. In 2012 in the UK, new diagnoses among MSM continued to rise, reaching an all-time high in 2012, with black African men and women being the second largest group affected by HIV [33]. HIV infection is an important issue in these communities and needs to be addressed directly within and by them. Although being a member of such a community does not mean that one is HIV-infected, individuals in these communities do have a higher chance of acquiring, living with, and transmitting HIV.

One way to reduce potential stigma or discrimination for such populations is to link HIV testing to other health checks, such as those for hypertension, diabetes or other conditions, thereby removing a direct link with HIV. This has been done in some middle-income countries [34]. For such linkage to occur, individuals in key populations must have access to relevant services and prevention technologies and knowledge of how to apply them. The relevant services, including facility-based services as well as outreach and community-based services, must have adequate resources and should be ‘stigma and discrimination free.’ In the UK, significant improvement in HIV test coverage in health facilities is needed: for instance, 29% of sexual health facility attendees in the UK in 2012 did not have an HIV test [33]. Expansion of HIV test coverage needs to occur within wider regional and national contexts in which stigma and discrimination towards the communities that drive the UK HIV epidemic are eliminated while the required resources, including political will, are deployed for a comprehensive response.

Without increased HIV testing, HIV will continue to spread in key populations in the UK and other countries, increasing the number of PLHIV, and necessitating ongoing expansion of HIV services. With a growing number of people projected to receive ART and benefit from prolonged life expectancy [35], the incidence of non-HIV comorbidities are also likely to increase [36], all putting additional demands on an already over-strained UK National Health Service [37] and health care systems in other high-income countries [28]. Though economic conditions will be different in middle- and lower-income countries, our findings may also be applicable to some of these countries [34].

## Supporting Information

Figure S1 **Schematic of HIV transmission and progression model.**
(DOCX)
