# Supplementary material for: Expanded HIV Testing in Low-Prevalence, High-Income Countries: A Cost-Effectiveness Analysis for the United Kingdom
Source: PLoS One. 2014 Apr 24;9(4):e95735. doi: 10.1371/journal.pone.0095735 (PMC3998955; doi:10.1371/journal.pone.0095735)
Supplement: Figure S1 — Schematic of HIV transmission and progression model. (DOCX) [file pone.0095735.s001.docx]

**Figure S1.** Schematic of HIV transmission and progression model. Although not shown in the figure, men are also distinguished in the model by circumcision status.
